# RIPK1 in Liver Parenchymal Cells Limits Murine Hepatitis during Acute CCl_4_-Induced Liver Injury

**DOI:** 10.3390/ijms23137367

**Published:** 2022-07-01

**Authors:** Huma Hameed, Muhammad Farooq, Céline Vuillier, Claire Piquet-Pellorce, Annaïg Hamon, Marie-Thérèse Dimanche-Boitrel, Michel Samson, Jacques Le Seyec

**Affiliations:** 1 Univ Rennes, Inserm, EHESP, IRSET (Institut de Recherche en Santé, Environnement ET Travail)–UMR_S 1085, F-35000 Rennes, France; huma4748@gmail.com (H.H.); muhammad.farooq@uvas.edu.pk (M.F.); vuillierceline@gmail.com (C.V.); claire.piquet-pellorce@univ-rennes1.fr (C.P.-P.); annaig.hamon@gmail.com (A.H.); marie-therese.boitrel@univ-rennes1.fr (M.-T.D.-B.); jacques.leseyec@univ-rennes1.fr (J.L.S.); 2 Department of Clinical Sciences, College of Veterinary and Animal Sciences, Jhang 35200, Pakistan

**Keywords:** carbon tetrachloride, acute hepatitis, drug-induced liver injury, receptor-interacting protein kinase-1, apoptosis

## Abstract

Some life-threatening acute hepatitis originates from drug-induced liver injury (DILI). Carbon tetrachloride (CCl_4_)-induced acute liver injury in mice is the widely used model of choice to study acute DILI, which pathogenesis involves a complex interplay of oxidative stress, necrosis, and apoptosis. Since the receptor interacting protein kinase-1 (RIPK1) is able to direct cell fate towards survival or death, it may potentially affect the pathological process of xenobiotic-induced liver damage. Two different mouse lines, either deficient for *Ripk1* specifically in liver parenchymal cells (*Ripk1*^LPC-KO^) or for the kinase activity of RIPK1 (*Ripk1*^K45A^, kinase dead), plus their respective wild-type littermates (*Ripk1*^fl/fl^, *Ripk1*^wt/wt^), were exposed to single toxic doses of CCl_4_. This exposure led in similar injury in *Ripk1*^K45A^ mice and their littermate controls. However, *Ripk1*^LPC-KO^ mice developed more severe symptoms with massive hepatocyte apoptosis as compared to their littermate controls. A pretreatment with a TNF-α receptor decoy exacerbated liver apoptosis in both *Ripk1*^fl/fl^ and *Ripk1*^LPC-KO^ mice. Besides, a FasL antagonist promoted hepatocyte apoptosis in *Ripk1*^fl/fl^ mice but reduced it in *Ripk1*^LPC-KO^ mice. Thus, the scaffolding properties of RIPK1 protect hepatocytes from apoptosis during CCl_4_ intoxication. TNF-α and FasL emerged as factors promoting hepatocyte survival. These protective effects appeared to be independent of RIPK1, at least in part, for TNF-α, but dependent on RIPK1 for FasL. These new data complete the deciphering of the molecular mechanisms involved in DILI in the context of research on their prevention or cure.

## 1. Introduction

Over the past 20 years, there has been a steady increase in research on drug-induced liver injury (DILI) [1]. This stems not only from its high prevalence observed in the diagnosis of acute liver failure [2], but also to risks of drug withdrawal from the pharmaceutical market. Two types of DILI are distinguished. Thus, the intrinsic DILI is directly related to the physicochemical properties of drugs, is dose-dependent, and largely predictable. Conversely, in rare cases, idiosyncratic DILI (IDILI) occurs unpredictably because it is specific to individuals, their environment, and possible comorbidities [3]. After the specific acetaminophen-poisoning animal model, the most popular in vivo model to study intrinsic DILI is the carbon tetrachloride (CCl_4_)-induced acute hepatitis [4]. CCl_4_ is an organic compound which was previously widely used as a precursor to refrigerants, cleaning agent, degreaser, and as a constituent in fire extinguishers until its hepatotoxic and carcinogenic potential was elucidated. Some clinical cases of intentional or accidental CCl_4_ intoxication have been reported as early as the beginning of the last century with examples still occurring until recently [5,6]. Xenobiotic metabolism enzymes, in particular cytochromes P450, transform CCl_4_ into the reactive metabolite trichloromethyl radical (CCl_3_•). This generated free radical alkylates nucleic acids, proteins, and lipids but reacts also with oxygen molecules to form trichloromethylperoxy radical (CCl_3_OO•). This highly reactive species then promotes lipid peroxidation. This combination of events results in hepatotoxicity [7,8].

CCl_4_ at high doses induce hepatocyte death, which can be necrotic or apoptotic depending upon activation of different signalling pathways [9]. In hepatocyte, reactive metabolites trigger endoplasmic reticulum or mitochondrial stress. In case of overwhelmed organelle-specific adaptive stress responses, hepatocyte death occurs either by mitochondrial permeability transition (MPT) leading to necrosis or by regulated cell death mechanisms leading to mitochondrial outer membrane permeabilization (MOMP), which initiates the intrinsic apoptotic pathway [10,11]. In case of extensive liver damage, it may be difficult to differentiate the type of cell death by histology [12]. Cell swelling and plasma membrane rupturing (oncosis), which promotes an inflammatory response, is defined as necrosis, while all combined, caspase activation, chromatin fragmentation, and phagocytosis of apoptotic cell bodies that reduces inflammation, define apoptosis [13,14]. Cell death then promotes a pro-inflammatory environment where the tumor necrosis factor (TNF-α) plays a key role. The intracellular signalling pathways, prompted by the interaction of TNF-α with its cognate receptors (TNFR1 and TNFR2), are governed by the receptor-interacting protein kinase-1 (RIPK1) [15]. Thus, the scaffolding properties of RIPK1 direct signal transduction to an NF-κB dependent pro-survival pathway, while its kinase activity directs the cell to death by apoptosis or by necroptosis [16]. Thus, some works demonstrated the protective role of RIPK1 in hepatocytes during different acute hepatitis involving TNF-α [17,18,19]. Regarding CCl_4_-induced hepatotoxicity, some studies focused on the implication of TNF-α [20,21], none investigate RIPK1. With two different mouse lines, either deficient for *Ripk1* specifically in liver parenchymal cells (*Ripk1*^LPC-KO^), or for the kinase activity of RIPK1 (*Ripk1*^K45A^) plus their respective WT littermates (*Ripk1*^fl/fl^ and *Ripk1^wt/wt^*), we aimed to deepen the role of RIPK1 during CCl_4_-induced hepatotoxicity.

## 2. Results

### 2.1. Scaffolding Properties of RIPK1 in Liver Parenchymal Cell Limit Apoptosis Occurrence in Acute CCl_4_-Induced Hepatitis

**Figure 1:**
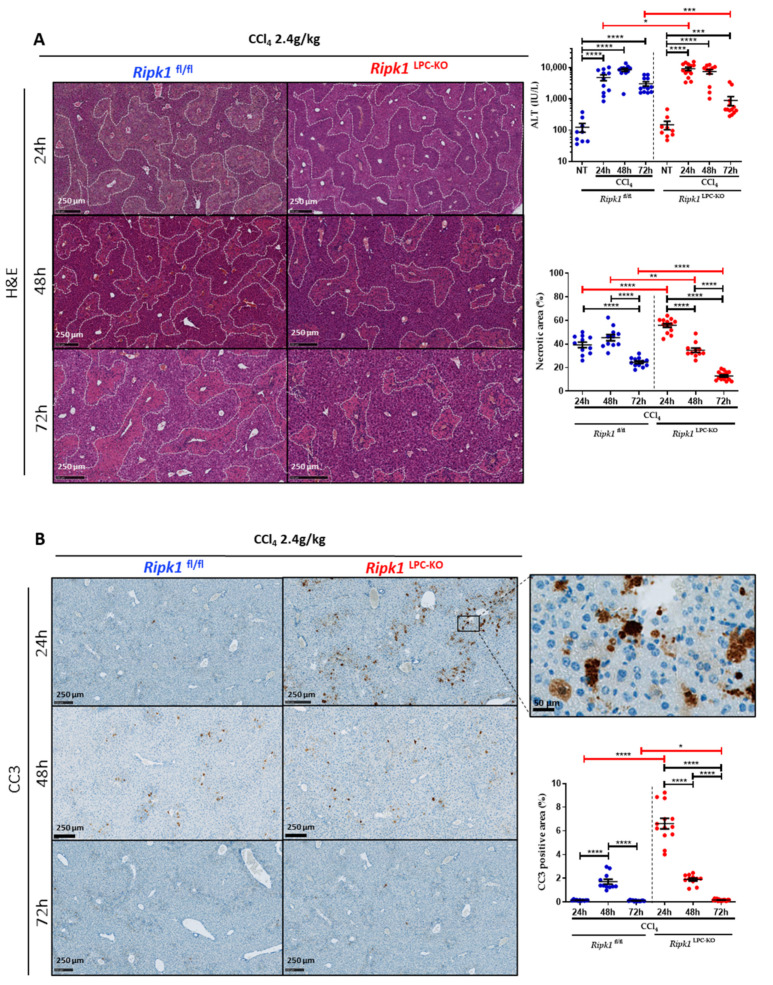
**RIPK1 helps protect liver parenchymal cells from apoptosis during CCl_4_ poisoning.***Ripk1*^fl/fl^ and *Ripk1*^LPC-KO^ mice were force-fed either with olive oil alone (control; NT for non-treated) or containing CCl_4_ (dose of 2.4 g/kg body weight). Sample collections were conducted 24, 48 or 72 h post-gavage. (**A**) Representative pictures of liver tissue sections stained by H&E with quantification of necrotic areas. Levels of serum alanine aminotransferase (ALT). (**B**) Representative pictures of liver tissue sections stained by cleaved caspase 3 (CC3) antibody. Signal quantification of CC3 staining. For all graphs, each dot represents an individual and errors bars are expressed as means ± SEM (* *p* < 0.05; ** *p* < 0.01; *** *p* < 0.001 and **** *p* < 0.0001).

To investigate the role of RIPK1 in CCl_4_-induced hepatotoxicity, *Ripk1*^LPC-KO^ mice along with their WT littermates (*Ripk1*^fl/fl^) were first exposed to a toxic dose of CCl_4_ by gavage. Liver damage was assessed by histopathological tissue staining and by serum transaminase measurement at 24, 48, and 72 h post-treatment (Figure 1A). In both genotypes, CCl_4_ treatment induced classical centrilobular liver damage as shown by H&E staining. The surface of necrotic areas appeared significantly larger in the livers of *Ripk1*^LPC-KO^ mice 24 h after the gavage. Besides, serum transaminase levels (ALT) significantly increased at 24 and 48 h and started to drop at 72 h. Even if the kinetics appeared similar between genotypes, the levels of serum transaminases reached significantly higher values in *Ripk1*^LPC-KO^ mice in samples taken at 24 h. On the other hand, samples taken 72 h after CCl_4_ administration revealed lower levels of necrotic areas and of serum transaminases in *Ripk1*^LPC-KO^ mice than in *Ripk1*^fl/fl^ littermates. This is most probably due to the earlier hepatocyte death in challenged *Ripk1*^LPC-KO^ mice. Interestingly, labelling of cleaved caspase-3 (CC3) showed that much more apoptosis occurred 24 h post-treatment in the livers of *Ripk1*^LPC-KO^ mice than in those of *Ripk1*^fl/fl^ littermates (Figure 1B).

**Figure 2:**
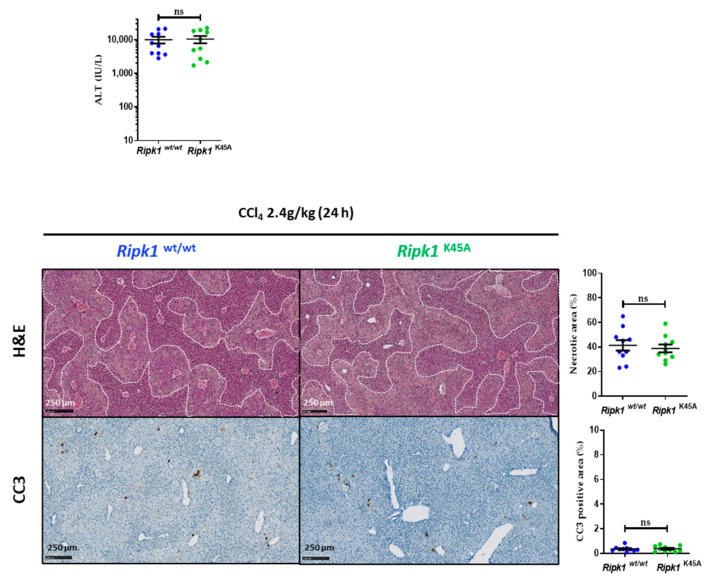
**No contribution of RIPK1 kinase activity during CCl_4_-induced hepatotoxicity.***Ripk1*^wt/wt^ and *Ripk1*^K45A^ mice were force-fed with olive oil containing CCl_4_ (dose of 2.4 g/kg body weight), 24 h before sample collection. Levels of serum alanine aminotransferase (ALT). Representative pictures of liver tissue sections stained by H&E or by cleaved caspase 3 (CC3) antibody with their respective quantifications. For all graphs, each dot represents an individual and errors bars are expressed as means ± SEM (ns, non-significant).

Next, mice deficient for the kinase activity of RIPK1 (*Ripk1*^K45A^) along with their WT littermates (*Ripk1*^wt/wt^) were subjected to the same CCl_4_ treatment. Similar liver injury occurred at 24 h post-treatment in both genotypes as shown by the histological analysis of livers and by the determination of serum transaminase levels (Figure 2). CC3 staining also showed no modification in apoptosis occurrence. Therefore, only the scaffolding properties of RIPK1 in liver parenchymal cells interfere with CCl_4_-hepatoxicity by avoiding the induction of extensive apoptosis.

### 2.2. Ripk1 Deficiency in Liver Parenchymal Cells Potentiates Oxidative Stress, Inflammation and Immune Infiltration in CCl_4_-Induced Hepatitis

**Figure 3:**
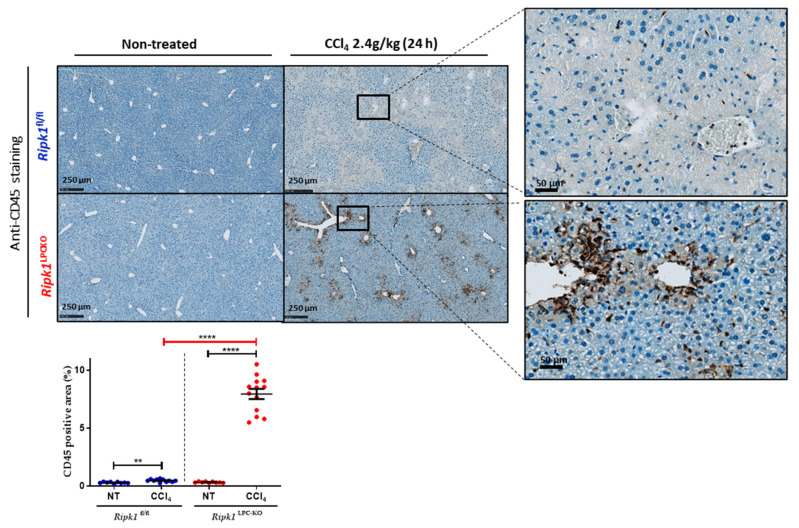
**Immune infiltration analysis in non-treated and CCl_4_-treated *Ripk1*^fl/fl^ and *Ripk1*^LPC-KO^ mice.***Ripk1*^fl/fl^ and *Ripk1*^LPC-KO^ mice were force-fed either with olive oil alone (control; NT for non-treated) or containing CCl_4_ (dose of 2.4 g/kg body weight), 24 h before sample collection. Representative pictures of liver tissue sections stained by an anti-CD45 antibody. Signal quantification of anti-CD45 positive area. For the graph, each dot represents an individual and errors bars are expressed as means ± SEM (** *p* < 0.01 and **** *p* < 0.0001).

**Figure 4:**
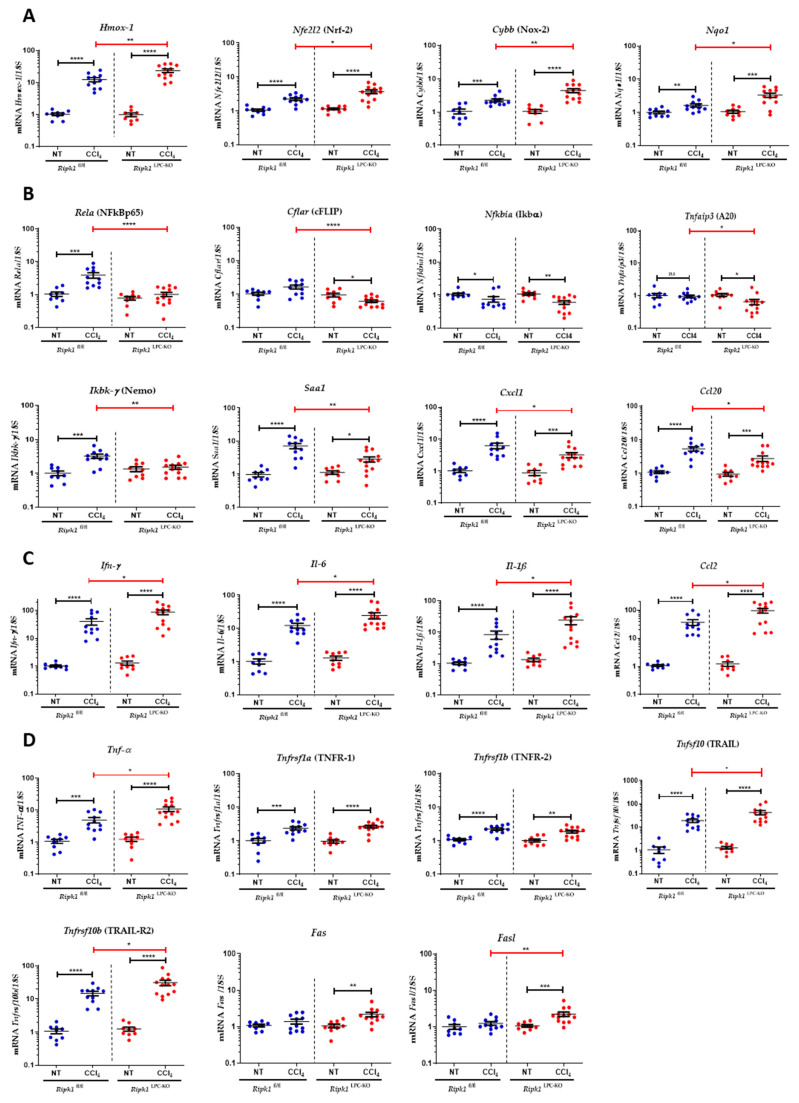
**Analysis of mRNA amounts of genes involved in oxidative stress or inflammation in the liver of non-treated and CCl_4_-treated *Ripk1*^fl/fl^ and *Ripk1*^LPC-KO^ mice.** *Ripk1*^fl/fl^ and *Ripk1*^LPC-KO^ mice were force-fed either with olive oil alone (control; NT for non-treated) or containing CCl_4_ (dose of 2.4 g/kg body weight) 24 h before sample collection. Hepatic mRNA expression levels of oxidative stress related genes (*Hmox*-*1*, *Cybb* [Nox-2], *Nfe2l2* [Nrf-2], and *Nqo1*) (**A**), of NF-κB related genes (*Rela* [NFkBp65], *Cflar* [cFLIP], *Nfkbia* [*I*κ*b*α], *Tnfaip3* [*A20*]), *Ikk-γ* [*NEMO*], *Saa1, Cxcl1, and Ccl20*) (**B**), of inflammation related genes (*Ifn*-*γ, Il*-*6, Il*-*1β,* and *Ccl2),* (**C**) and of death factor genes and their cognate receptors (*Tnf*-*α, Tnfrsf1a* [*TNFR*-*1*], *Tnfrsf1b* [*TNFR*-*2*], *Tnfsf10* [*TRAIL*], *Tnfrsf10b* [*TRAIL*-*R2*], *Fas, and Fasl*) (**D**). For all graphs, each dot represents an individual and errors bars are expressed as means ± SEM (ns, non-significant; * *p* < 0.05; ** *p* < 0.01; *** *p* < 0.001, and **** *p* < 0.0001).

CCl_4_ metabolism triggers the production of reactive oxygen species (ROS), which contribute to cell toxicity and ultimately result in the release of damage-associated molecular patterns (DAMPs) into the extracellular environment, a key aspect of inflammation that further activate Kupffer cells (KCs), monocyte-derived macrophages (MFs) and neutrophils. These activated immune cells will then release pro-inflammatory cytokines in liver that will exacerbate cell damage. To investigate these different features of the pathological process, the analysis of immune infiltration and the regulation of mRNA levels of several genes was compared in the livers of *Ripk1*^fl/fl^ and *Ripk1*^LPC-KO^ mice, untreated for 24 h after CCl_4_ gavage. Thus, the analysis of immune infiltration by CD45 staining revealed much more infiltrated leukocytes in the hepatic tissue of CCl_4_-treated *Ripk1*^LPC-KO^ mice (Figure 3). The specific analysis of the macrophage subpopulation (CD68+ cells) revealed a similar but less marked trend (Figure S1). Accordingly, all tested mRNA levels of genes related to oxidative stress (*Hmox-1*, *Nfe2l2* [Nrf-2], *Cybb* [Nox-2], and *Nqo1*) were more induced by CCl_4_ treatment in *Ripk1*^LPC-KO^ mice (Figure 4A). Conversely, mRNA level inductions of evaluated genes related to the NF-κB pathway (*Rela* [NFkBp65], *Cflar* [cFLIP], *Tnfaip3* [*A20*], *Ikk-γ* [*NEMO*], *Saa1, Cxcl1, and Ccl20*) were significantly less pronounced in *Ripk1*^LPC-KO^ mouse samples (except for *Nfkbia* [*I*κ*b*α]) (Figure 4B). However, a similar analysis on mRNA of genes involved in inflammation (*Ifnγ, Il-6, Il-1β and Ccl2*) systematically showed an exacerbated induction in the liver of *Ripk1*^LPC-KO^ mice after CCl_4_ gavage (Figure 4C). The same expression regulation of these cytokines was found at the protein level during their serum quantification (Figure S2). These results supported the notion that the scaffolding properties of RIPK1 would protect hepatocytes from apoptosis during CCl_4_ intoxication by activating the NF-κB pathway. The higher rate of hepatocyte mortality observed in *Ripk1*^LPC-KO^ mice is thus thought to be the cause of exacerbated oxidative stress, inflammation, and immune infiltration. In parallel, mRNA levels of death factors and their cognate receptors were also investigated in livers. Thus, the mRNA amounts for *Tnfrsf1a* [*TNFR-1*] and *Tnfrsf1b* [*TNFR-2*] were higher after CCl_4_ gavage, but with similar induction rates regardless of the genotype. For the mRNA of *Tnf-α, Tnfsf10* [*TRAIL*] and *Tnfrsf10b* [*TRAIL-R2*], the induction appeared more important in *Ripk1*^LPC-KO^ mice. Finally, slight mRNA inductions were detected only in challenged *Ripk1*^LPC-KO^ mice for the FasL/Fas pair (Figure 4D).

### 2.3. Neutralization of TNF-α Further Potentiates CCl_4_-Induced Hepatotoxicity in Ripk1^fl/fl^ and Ripk1^LPC-KO^ Mice

**Figure 5:**
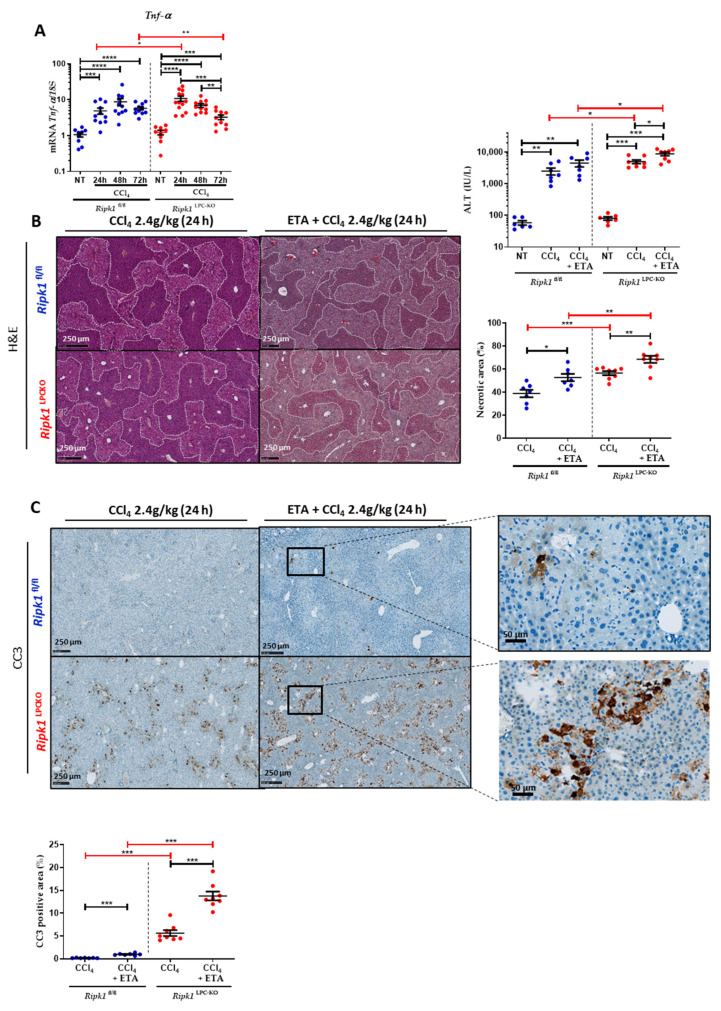
**Neutralization of TNF-α further potentiated CCl_4_-induced apoptosis.***Ripk1*^fl/fl^ and *Ripk1*^LPC-KO^ mice were force-fed either with olive oil alone (control; NT for non-treated) or containing CCl_4_ (dose of 2.4 g/kg body weight), with eventual treatments with etanercept (ETA, 10 mg/kg, 1 h before and 12 h after gavage), 24 h before sample collection. (**A**) Hepatic mRNA expression levels of *Tnf-α*. (**B**) Levels of serum alanine aminotransferase (ALT). Representative pictures of liver tissue sections stained by H&E with quantification of necrotic areas. (**C**) Representative pictures of liver tissue sections stained by cleaved caspase 3 (CC3) antibody. Signal quantification of CC3 staining. For all graphs, each dot represents an individual and errors bars are expressed as means ± SEM. (* *p* < 0.05; ** *p* < 0.01; *** *p* < 0.001, and **** *p* < 0.0001).

**Figure 6:**
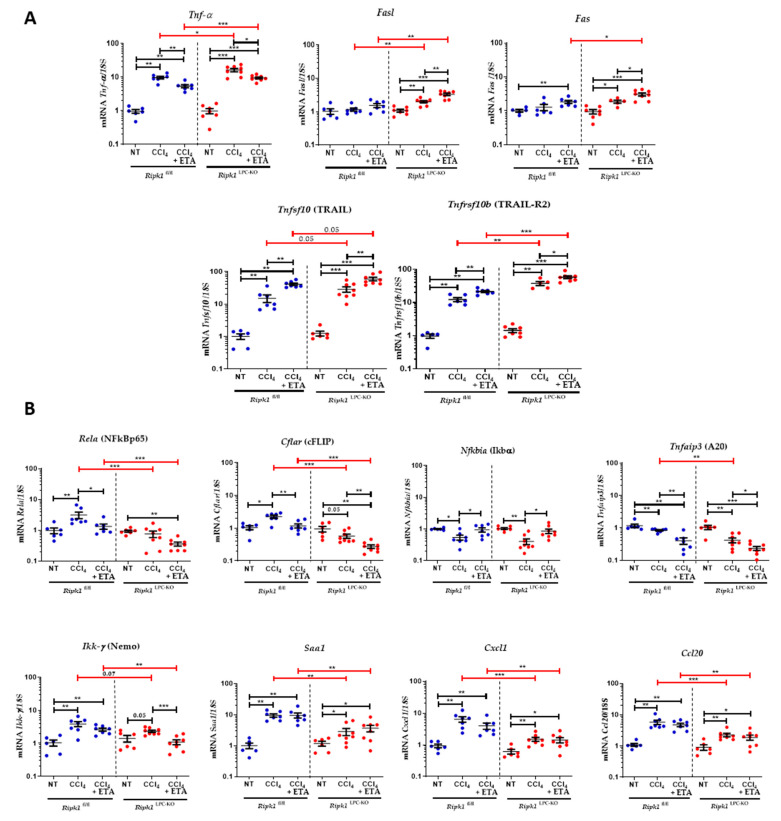
Analysis of mRNA amounts of genes involved in inflammation in the liver of ETA-CCl_4_ treated *Ripk1*^fl/fl^ and *Ripk1*^LPC-KO^ mice. *Ripk1*^fl/fl^ and *Ripk1*^LPC-KO^ mice were force-fed either with olive oil alone (control; NT for non-treated) or containing CCl_4_ (dose of 2.4 g/kg body weight), with eventual treatments with etanercept (ETA, 10 mg/kg, 1 h before and 12 h after gavage), 24 h before sample collection. (**A**) Hepatic mRNA expression levels *Tnf-α*, *Fasl*, *Fas*, *Tnfsf10* [*TRAIL*], and *Tnfsf10b* [*TRAIL-R2*]. (**B**) Hepatic mRNA expression levels of *Rela* [NFkBp65], *Cflar* [cFLIP], *Nfkbia* [*I*κ*b*α], *Tnfaip3* [*A20*], *Ikk-γ* [*NEMO*], *Saa1, Cxcl1,* and *Cccl20.* For all graphs, each dot represents an individual and errors bars are expressed as means ±SEM. (* *p* < 0.05; ** *p* < 0.01; and *** *p* < 0.001).

To continue the investigations on the potential role of TNF-α during CCl_4_-induced hepatitis, levels of its mRNA were followed at 24, 48, and 72 h post-gavage and compared to levels found in untreated mice. The induction peaks were observed in the livers of CCl_4_-treated *Ripk1*^LPC-KO^ mice at 24 h post-treatment and at 48 h post-treatment for *Ripk1*^fl/fl^ mice (Figure 5A). To then assess the involvement of TNF-α in the pathophysiology of CCl_4_-induced acute hepatitis, etanercept (ETA) was administered 1 h prior CCl_4_ gavage and 12 h later (Figure S3). Administration of this TNF-α decoy receptor aggravated liver damage in both *Ripk1*^fl/fl^ and *Ripk1*^LPC-KO^ mice as shown by the assessment of necrotic surfaces in the liver tissue and by the quantification of serum transaminases on samples collected 24 h after the gavage (Figure 5B). As already described above (Figure 2), cleaved caspase-3 staining of hepatic tissue sections revealed more apoptotic cells after CCl_4_ treatment in the liver of *Ripk1*^LPC-KO^ mice, but the addition of ETA significantly increased the proportion of affected cells for both genotypes (Figure 5C). The mRNA levels of different genes were then assessed in the liver of *Ripk1*^fl/fl^ and *Ripk1*^LPC-KO^ mice under the various experimental conditions. Thus, adding ETA to CCl_4_ treatment partially limited the induction of *Tnf*-α mRNA levels. Interestingly, quantities of *Fasl, Fas*, *Tnfsf10* [*TRAIL*], *and Tnfrsf10b* [*TRAIL-R2*] mRNAs were over-induced especially in *Ripk1*^LPC-KO^ in presence of ETA, which correlated with the worsening of hepatitis (Figure 6A). Concerning tested genes involved in the NF-κB survival signalling pathway, while for *Saa1, Cxcl1,* and *Ccl20,* no change occurred in presence of ETA, the TNF-α decoy receptor lowered the levels of the other studied mRNAs, i.e., *Rela* [NFkBp65], *Cflar* [cFLIP], *Tnfaip3* [*A20*], and *Ikk-γ* [*NEMO*] (except for *Nfkbia* [*I*κ*b*α]) (Figure 6A) for both genotypes. Together, these observations suggested that the overall pro-inflammatory effect of NF-κB was decreased in both *Ripk1*^fl/fl^ and *Ripk1*^LPC-KO^ by the addition of ETA. These later observations meant that in acute CCl_4_-induced hepatitis, TNF-α would help protect hepatocytes through both RIPK1-independent and dependent mechanisms.

### 2.4. Neutralization of FasL Partially Increased CCl_4_-Induced Hepatotoxicity in Ripk1^LPC-KO^

**Figure 7:**
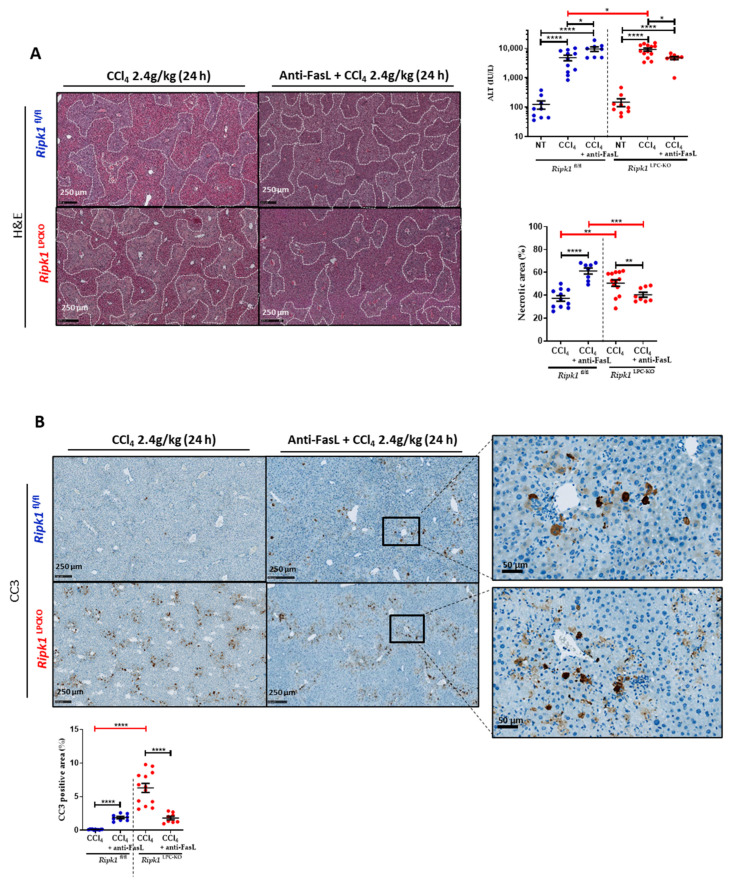
**Neutralization of FasL impacted CCl_4_-induced hepatotoxicity.***Ripk1*^fl/fl^ and *Ripk1*^LPC-KO^ mice were force-fed either with olive oil alone (control; NT for non-treated) or containing CCl_4_ (dose of 2.4 g/kg body weight), with an eventual pre-treatment with an anti-FasL antibody (1 h before gavage), 24 h before sample collection. (**A**) Levels of serum alanine aminotransferase (ALT). Representative pictures of liver tissue sections stained by H&E with quantification of necrotic areas. (**B**) Representative pictures of liver tissue sections stained by cleaved caspase 3 (CC3) antibody. Signal quantification of CC3 staining. For all graphs, each dot represents an individual and errors bars are expressed as means ± SEM. (* *p* < 0.05; ** *p* < 0.01; *** *p* < 0.001; and **** *p* < 0.0001).

**Figure 8:**
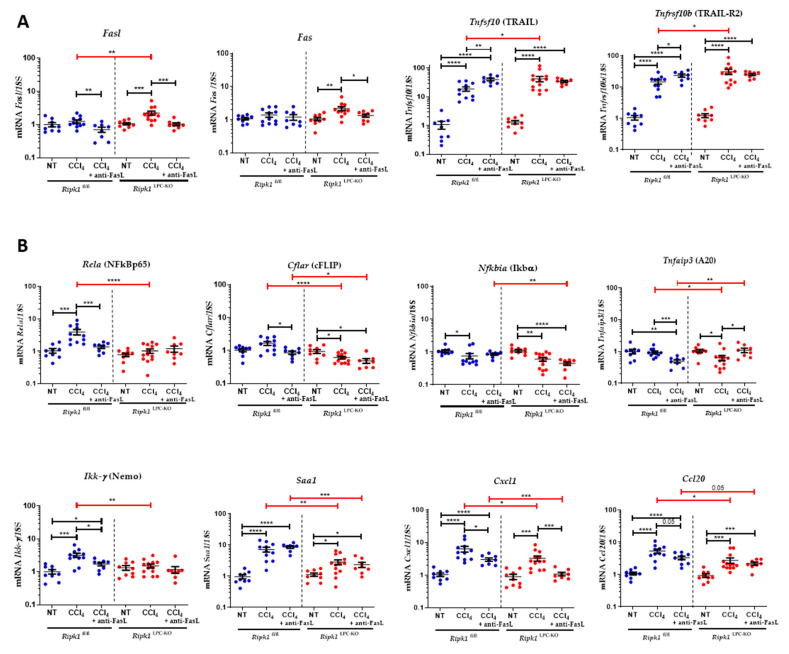
Analysis of mRNA amounts of genes involved in inflammation in the liver of anti-FasL-CCl_4_ treated *Ripk1*^fl/fl^ and *Ripk1*^LPC-KO^ mice. *Ripk1*^fl/fl^ and *Ripk1*^LPC-KO^ mice were force-fed either with olive oil alone (control; NT for non-treated) or containing CCl_4_ (dose of 2.4 g/kg body weight), with an eventual pre-treatment with an anti-FasL antibody (1 h before gavage), 24 h before sample collection. (**A**) Hepatic mRNA expression levels of *Fasl*, *Fas*, *Tnfsf10* [*TRAIL*], *and Tnfrsf10b* [*TRAIL-R2*]. (**B**) Hepatic mRNA expression levels of *Rela* [*NFkBp65*], *Cflar* [*cFLIP*], *Nfkbia* [*I*κ*b*α], *Tnfaip3* [*A20*], *Ikk-γ* [*NEMO*], *Saa1, Cxcl1,* and *Ccl20*. For all graphs, each dot represents an individual and errors bars are expressed as means ±SEM. (* *p* < 0.05; ** *p* < 0.01; *** *p* < 0.001; and **** *p* < 0.0001).

Based on above results, we further investigated the involvement of FasL in CCl_4_-mediated hepatitis. Experiments were conducted on *Ripk1*^fl/fl^ and *Ripk1*^LPC-KO^ mice undergoing CCl_4_-induced acute hepatitis with or without a pre-treatment with a FasL antagonist intraperitoneally administrated 1 h before CCl_4_ gavage. Liver damage was analysed 24 h post-administration of CCl_4_. While the FasL antagonist worsened the hepatic injury in *Ripk1*^fl/fl^ mice as evidenced by H&E staining of liver sections and serum transaminase level measurements (Figure 7A), the anti-FasL antibody had a partial protective effect in *Ripk1*^LPC-KO^ mice. These opposite effects were also found when evaluating the rates of cell death by apoptosis occurring in liver tissue (Figure 7B). Thus, during CCl_4_ acute hepatitis, FasL appeared to participate to the protection of hepatocytes when these cells are not modified and expressed RIPK1, while its absence in liver parenchymal cells sensitize them to the pro-death function of FasL. The mRNA levels of different genes (*Fasl*, *Fas*, *Tnfsf10* [*TRAIL*], *Tnfrsf10b* [*TRAIL-R2*], *Rela* [*NFkBp65*], *Cflar* [cFLIP], *Nfkbia* [*IκBα*], *Tnfaip3* [*A20*], *Ikk-γ* [*NEMO*], *Saa1, Cxcl1,* and *Ccl20*) were then assessed in the liver of *Ripk1*^fl/fl^ and *Ripk1*^LPC-KO^ mice under the various experimental conditions (Figure 8). Thus, adding anti-FasL to CCl_4_ treatment partially limited the induction of *FasL* mRNA levels. Interestingly, quantities of *Tnfsf10* [*TRAIL*] and *Tnfrsf10b* [*TRAIL-R2*] mRNAs were over-induced in *Ripk1*^fl/fl^ in presence of anti-FasL, correlating with symptom worsening (Figure 8A). Concerning tested genes involved in the NF-κB survival signalling pathway, their mRNA levels generally tended to drop in *Ripk1*^fl/fl^ and *Ripk1*^LPC-KO^ mice when adding the anti-FasL (Figure 8B). These observations suggested that FasL plays a protective role in CCl_4_ induced acute hepatitis, potentially by promoting the NF-κB signalling pathway.

## 3. Discussion

Downstream of different death ligand receptors, RIPK1 has a pro-survival function through its scaffolding properties or it triggers cell death through its kinase activity [22]. Thus, RIPK1 can induce caspase-dependent apoptosis, or necroptosis by coordinating with RIPK3 and the mixed lineage kinase domain like pseudokinase (MLKL) [23]. We recently demonstrated the protective role of RIPK1 in NASH induced fibrosis [24]. In parallel, RIPK1 interaction with RIPK3 has been described to increase with repeated injections of CCl_4_, suggesting the possible involvement of RIPK1 in chronic CCl_4_-induced hepatotoxicity [25]. In contrast to the chronic model of CCl_4_-induced hepatitis obtained by repeated expositions which is more focused on liver fibrosis pathophysiology [26], our investigations covered the acute CCl_4_ liver toxicity after single dose administration. Thus, we demonstrated that RIPK1 depletion specifically in liver parenchymal cells sensitizes mice (*Ripk1*^LPC-KO^) to CCl_4_-induced hepatocyte apoptosis, while genetic inactivation of its kinase activity (*Ripk1*^K45A^) never affected CCl_4_-induced acute hepatitis. Further, pharmacological inhibition of RIPK1 by Nec1s also had no impact on CCl_4_-induced hepatitis in mice (Figure S4). Therefore, the involvement of RIPK1 in CCl_4_-induced acute hepatitis engages its platform function but not its kinase activity. Thus, as in the specific case of acetaminophen intoxication [27], the kinase activity of RIPK1 does not represent a relevant therapeutic target.

CCl_4_-induced hepatotoxicity has been associated with increased expression of hepatic transcript and of plasma concentrations of TNF-α [20,28]. Besides, RIPK1 is known to play a protective role in different type of acute hepatitis involving TNF-α [17,18,19]. Although the TNF signalling pathway has been depicted as not critical for chronic exposure to CCl_4_ [21], the cytokine is believed to contribute to the damage occurring in acute CCl_4_-induced hepatitis. Indeed, its neutralization or depletion of its receptor (TNFR1) has been described as leading to an improvement of clinical signs in animal models [28]. However, our experiments using a TNF-α decoy receptor (ETA) did not reproduce this protection. On the contrary, apoptosis was over-induced in the livers of *Ripk1*^fl/fl^ control mice as well as in those of *Ripk1*^LPC-KO^ mice in presence of ETA. This protective effect of TNF-α appeared, therefore, at least in part, independent of RIPK1 present in liver parenchymal cells, without excluding its potential involvement in other cell types present in the hepatic tissue. This limitation of the deleterious effects of CCl_4_ by TNF-α has been also reported in acute intoxication in rats [20]. However, its role emerged as more complex [20]. Indeed, if low ETA concentrations limited liver injury caused by CCl_4_ in rats, higher concentrations lost the protective effect and can even aggravate symptoms. Altogether, these different data probably reflect the coexistence of the protective and harmful properties of TNF-α in acute hepatitis induced by CCl_4_, a balance which could be influence depending on the experimental conditions.

The increased susceptibility of *Ripk1*^LPC-KO^ mice to acute hepatitis induced by CCl_4_, which was amplified in presence of ETA, was systematically associated with proportional mRNA upregulation of FasL/Fas couple in the liver. The ligand, FasL, exists in transmembrane or soluble form. Although both bind efficiently to the cognate receptor (Fas), interaction with the soluble form triggers rapid internalization of the complex, leading to downregulation of the Fas receptor on the cell surface [29,30]. Therefore, the soluble FasL compete with its membrane-bound counterpart for apoptosis induction. Thus, the conversion of the cytotoxic membrane-bound FasL to its protective soluble form has been proposed to represent an important regulatory mechanism by which susceptible tissues, such as the liver, protect themselves from excessive immune responses associated with infiltrating cytotoxic T cells harboring transmembrane FasL [30]. During our investigations, we found that an antagonist of FasL (anti-FasL antibody) interfered with the severity of CCl_4_-induced hepatitis. In control mice with the *Ripk1*^fl/fl^ genotype, the apoptotic death rate of hepatocytes increased, most probably reflecting the presence of a protective activity of FasL. Conversely, the same experiment conducted on *Ripk1*^LPC-KO^ mice revealed a predominant activity of FasL rather favorable to apoptosis induction. These results were consistent with previously published data showing a protective role of RIPK1 downstream of Fas in hepatocytes [31]. During our experimentations, it appeared that mRNA levels of TRAIL and TRAIL-R2 were upregulated during acute CCl_4_ poisoning, highlighting the interest of further investigating this TRAIL/TRAILR axis. Overall, this study provides a deeper understanding of mechanisms occurring during acute liver hepatitis induced by CCl_4_.

## 4. Materials and Methods

### 4.1. Animals, Treatment Protocols

Both mice lines, C57BL/6J *Ripk1*^LPC-KO^ and C57BL/6N *Ripk1*^K45A^ mice along with their respective WT littermates (*Ripk1*^fl/fl^ and *Ripk1*^wt/wt^) were maintained in our animal facility. Homogeneous groups of female and male mice aged 9 to 12 weeks were used for each experiment. CCl_4_ diluted in olive oil was administered orally at a dose of 2.4 g/kg body weight (10 μL/g body weight). Olive oil alone, administered in age and sex matched mice, was used as control. Etanercept (ETA, Pfizer, New-York, NY, USA) or anti-FasL antibody (Ultra-LEAF^TM^ anti-CD178 clone MFL3, Biolegend, Amsterdam, The Netherlands) were eventually injected via intraperitoneal route, both at a dose of 10 mg/kg body weight, 1 h prior CCl_4_ challenge and 12 h later only for ETA. Mice were euthanized at different indicated time points. A schematic diagram representing the experimental design is shown in Supplementary Data (Figure S3).

### 4.2. Biochemical Studies

Plasma alanine (ALT) and aspartate (AST) transaminases were measured according to the International Federation of Clinical Chemistry and Laboratory Medicine (IFCC) primary reference procedures using Olympus AU2700 Chemistry analyser^®^ (Olympus Optical, Tokyo, Japan). Only the ALT data are depicted since those of the AST systematically followed the same variations.

### 4.3. Histopathological Studies and Immunolocalization in Liver Tissues

Mouse liver samples were fixed in 4% paraformaldehyde and embedded in paraffin for immunohistochemistry. For histopathology, hematoxylin and eosin (H&E) staining of liver tissues was carried out to investigate the liver injury. For immunolocalization, paraffin-embedded mouse liver sections (5 µm) were dried for 1 h at 58 °C, followed by antigen retrieval and incubation with primary antibody (anti-cleaved caspase-3, Cell Signaling Technology, #9661, Danvers, MA, USA, or anti-CD45 antibody, BioLegend, #103107, Amsterdam, Netherlands) in a Ventana automated staining platform (Ventana Medical Systems, Illkirch-Graffenstaden, France). Revelation of primary antibody was carried out using horseradish peroxidase (HRP)-conjugated secondary antibody (Dako, Agilent Technologies, Les Ulis, France) and DAB substrate kit (Ventana Medical Systems, #760-124, Illkirch-Graffenstaden, France). Slides were then counterstained with hematoxylin. All paraffin-embedded liver sections were scanned with a digital slide scanner (Nanozoomer 2.0-RS, Hamamatsu Photonics, Massy, France) and files were analyzed with the NDP viewer 2.5 software (Hamamatsu, Hamamatsu City, Japan). Signal quantifications were performed with an image analysis software (NIS-Element AR analysis software, Nikon, Tokyo, Japan).

### 4.4. RNA Isolation and qPCR Analysis

Total RNA was extracted from frozen liver fragments using the Nucleospin RNA kit (Macherey-Nagel, #740955, Hoerdt, France) and an Ultra-Turrax^®^ homogenizer according to manufacturer’s instructions. After RNA quantification using the NanoDrop (ND-1000 Spectrophotometer, Thermo Fisher Scientific, Illkirch, France), cDNA was synthetized using the SuperScriptTM II Reverse Transcriptase (Thermo Fisher Scientific, #18064022, Illkirch, France). Real-time quantitative PCR was performed using the fluorescent SYBR Green dye (Power SYBR^®^ Green PCR Master Mix, Thermo Fisher Scientific, Illkirch, France) and the CFX384 Touch ^TM^ Real-Time PCR Detection System (Bio-Rad, Marnes-La-Coquette, France). Each measurement was performed in triplicate. The relative gene expression was normalized against the *18S* gene expression. Healthy control mice served as a reference for mRNA expression (control mRNA level was arbitrarily set at 1). All primer sequences are depicted in Table S1 in the supporting information.

### 4.5. Statistical Analysis

Data were expressed as means ± SEM for all mice treated similarly. Mean differences between experimental groups were assessed using the non-parametric Mann-Whitney U-test. All statistical analysis was achieved with the GraphPad Prism5 software. Significance is shown as follows: * *p* < 0.05, ** *p* < 0.01, *** *p* < 0.001, and **** *p* < 0.0001.

## Supplementary Materials

The following supporting information can be downloaded at: www.mdpi.com/article/10.3390/ijms23137367/s1.

## References

[1] Liang D., Guan Y., Zhu J., Wu J., Yu X., Qiu K., He Z., He Q. (2021). Global research trends of drug-induced liver injury (DILI) in the past two decades: A bibliometric and visualized study. Ann. Palliat. Med..

[2] Lee W.M. (2008). Etiologies of acute liver failure. Semin. Liver Dis..

[3] Andrade R.J., Aithal G.P., Björnsson E.S., Kaplowitz N., Kullak-Ublick G.A., Larrey D., Karlsen T.H. (2019). EASL Clinical Practice Guidelines: Drug-induced liver injury. J. Hepatol..

[4] McGill M.R., Jaeschke H. (2019). Animal models of drug-induced liver injury. Biochim. Biophys. Acta Mol. Basis Dis..

[5] Teschke R. (2018). Liver Injury by Carbon Tetrachloride Intoxication in 16 Patients Treated with Forced Ventilation to Accelerate Toxin Removal via the Lungs: A Clinical Report. Toxics.

[6] Meaden C.W., Procopio G., Calello D.P., Nelson L.S., Ruck B., Gupta A., Jacob J.E. (2020). Carbon tetrachloride poisoning from an antique fire extinguisher. Am. J. Emerg. Med..

[7] Boll M., Weber L.W., Becker E., Stampfl A. (2001). Mechanism of carbon tetrachloride-induced hepatotoxicity. Hepatocellular damage by reactive carbon tetrachloride metabolites. Z. Nat. C J. Biosci..

[8] Weber L.W., Boll M., Stampfl A. (2003). Hepatotoxicity and mechanism of action of haloalkanes: Carbon tetrachloride as a toxicological model. Crit. Rev. Toxicol..

[9] Dai C., Xiao X., Li D., Tun S., Wang Y., Velkov T., Tang S. (2018). Chloroquine ameliorates carbon tetrachloride-induced acute liver injury in mice via the concomitant inhibition of inflammation and induction of apoptosis. Cell Death Dis..

[10] Andrade R.J., Chalasani N., Björnsson E.S., Suzuki A., Kullak-Ublick G.A., Watkins P.B., Devarbhavi H., Merz M., Lucena M.I., Kaplowitz N. (2019). Drug-induced liver injury. Nat. Rev. Dis. Primers.

[11] Green D.R., Galluzzi L., Kroemer G. (2014). Cell biology. Metabolic control of cell death. Science.

[12] Shojaie L., Iorga A., Dara L. (2020). Cell Death in Liver Diseases: A Review. Int. J. Mol. Sci..

[13] Jaeschke H., Ramachandran A. (2020). Acetaminophen-induced apoptosis: Facts versus fiction. J. Clin. Transl. Res..

[14] Fink S.L., Cookson B.T. (2005). Apoptosis, pyroptosis, and necrosis: Mechanistic description of dead and dying eukaryotic cells. Infect. Immun..

[15] Gough P., Myles I.A. (2020). Tumor Necrosis Factor Receptors: Pleiotropic Signaling Complexes and Their Differential Effects. Front. Immunol..

[16] Ofengeim D., Yuan J. (2013). Regulation of RIP1 kinase signalling at the crossroads of inflammation and cell death. Nat. Rev. Mol. Cell Biol..

[17] Farooq M., Filliol A., Simoes Eugénio M., Piquet-Pellorce C., Dion S., Raguenes-Nicol C., Jan A., Dimanche-Boitrel M.T., Le Seyec J., Samson M. (2019). Depletion of RIPK1 in hepatocytes exacerbates liver damage in fulminant viral hepatitis. Cell Death Dis..

[18] Filliol A., Piquet-Pellorce C., Le Seyec J., Farooq M., Genet V., Lucas-Clerc C., Bertin J., Gough P.J., Dimanche-Boitrel M.T., Vandenabeele P. (2016). RIPK1 protects from TNF-α-mediated liver damage during hepatitis. Cell Death Dis..

[19] Filliol A., Piquet-Pellorce C., Raguénès-Nicol C., Dion S., Farooq M., Lucas-Clerc C., Vandenabeele P., Bertrand M.J.M., Le Seyec J., Samson M. (2017). RIPK1 protects hepatocytes from Kupffer cells-mediated TNF-induced apoptosis in mouse models of PAMP-induced hepatitis. J. Hepatol..

[20] Dong Y., Liu Y., Kou X., Jing Y., Sun K., Sheng D., Yu G., Yu D., Zhao Q., Zhao X. (2016). The protective or damaging effect of Tumor necrosis factor-α in acute liver injury is concentration-dependent. Cell Biosci..

[21] Simeonova P.P., Gallucci R.M., Hulderman T., Wilson R., Kommineni C., Rao M., Luster M.I. (2001). The role of tumor necrosis factor-alpha in liver toxicity, inflammation, and fibrosis induced by carbon tetrachloride. Toxicol. Appl. Pharmacol..

[22] Van T.M., Polykratis A., Straub B.K., Kondylis V., Papadopoulou N., Pasparakis M. (2017). Kinase-independent functions of RIPK1 regulate hepatocyte survival and liver carcinogenesis. J. Clin. Investig..

[23] Kondylis V., Pasparakis M. (2019). RIP Kinases in Liver Cell Death, Inflammation and Cancer. Trends Mol. Med..

[24] Farooq M., Simoes Eugénio M., Piquet-Pellorce C., Dion S., Raguenes-Nicol C., Santamaria K., Kara-Ali G.H., Larcher T., Dimanche-Boitrel M.-T., Samson M. (2022). Receptor-interacting protein kinase-1 ablation in liver parenchymal cells promotes liver fibrosis in murine NASH without affecting other symptoms. J. Mol. Med..

[25] Choi H.S., Kang J.W., Lee S.M. (2015). Melatonin attenuates carbon tetrachloride-induced liver fibrosis via inhibition of necroptosis. Transl. Res. J. Lab. Clin. Med..

[26] Scholten D., Trebicka J., Liedtke C., Weiskirchen R. (2015). The carbon tetrachloride model in mice. Lab. Anim..

[27] Hameed H., Farooq M., Piquet-Pellorce C., Hamon A., Samson M., Le Seyec J. (2022). Questioning the RIPK1 kinase activity involvement in acetaminophen-induced hepatotoxicity in mouse. Free Radic. Biol. Med..

[28] Morio L.A., Chiu H., Sprowles K.A., Zhou P., Heck D.E., Gordon M.K., Laskin D.L. (2001). Distinct roles of tumor necrosis factor-alpha and nitric oxide in acute liver injury induced by carbon tetrachloride in mice. Toxicol. Appl. Pharmacol..

[29] Guicciardi M.E., Gores G.J. (2006). Fasl and fas in liver homeostasis and hepatic injuries. Fas Signaling.

[30] Pinkoski M.J., Brunner T., Green D.R., Lin T. (2000). Fas and Fas ligand in gut and liver. Am. J. Physiol. Gastrointest. Liver Physiol..

[31] Filliol A., Farooq M., Piquet-Pellorce C., Genet V., Dimanche-Boitrel M.T., Vandenabeele P., Bertrand M.J.M., Samson M., Le Seyec J. (2017). RIPK1 protects hepatocytes from death in Fas-induced hepatitis. Sci. Rep..

