# Supplementary material for: RIPK1 in Liver Parenchymal Cells Limits Murine Hepatitis during Acute CCl4-Induced Liver Injury
Source: Int J Mol Sci. 2022 Jul 1;23(13):7367. doi: 10.3390/ijms23137367 (PMC9266426; doi:10.3390/ijms23137367)
Supplement: Supplementary file 1 [file ijms-23-07367-s001.zip › ijms-1686667-supplementary.pdf]

Figure S1

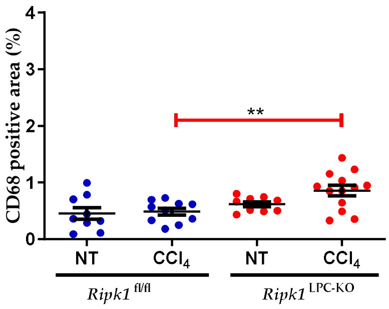

Figure S1: Macrophage infiltration analysis in non-treated and CCl_4-treated *Ripk1*$^{fl/fl}$ and *Ripk1*$^{LPC-KO}$ mice. *Ripk1*$^{fl/fl}$ and *Ripk1*$^{LPC-KO}$ mice were force-fed either with olive oil alone (control; NT for non-treated) or containing CCl_4 (dose of 2.4 g/kg body weight), 24 h before sample collection. Signal quantification of anti-CD68 positive area. For the graph, each dot represents an individual and errors bars are expressed as means ±SEM (** $p < 0.01$).

Figure S2

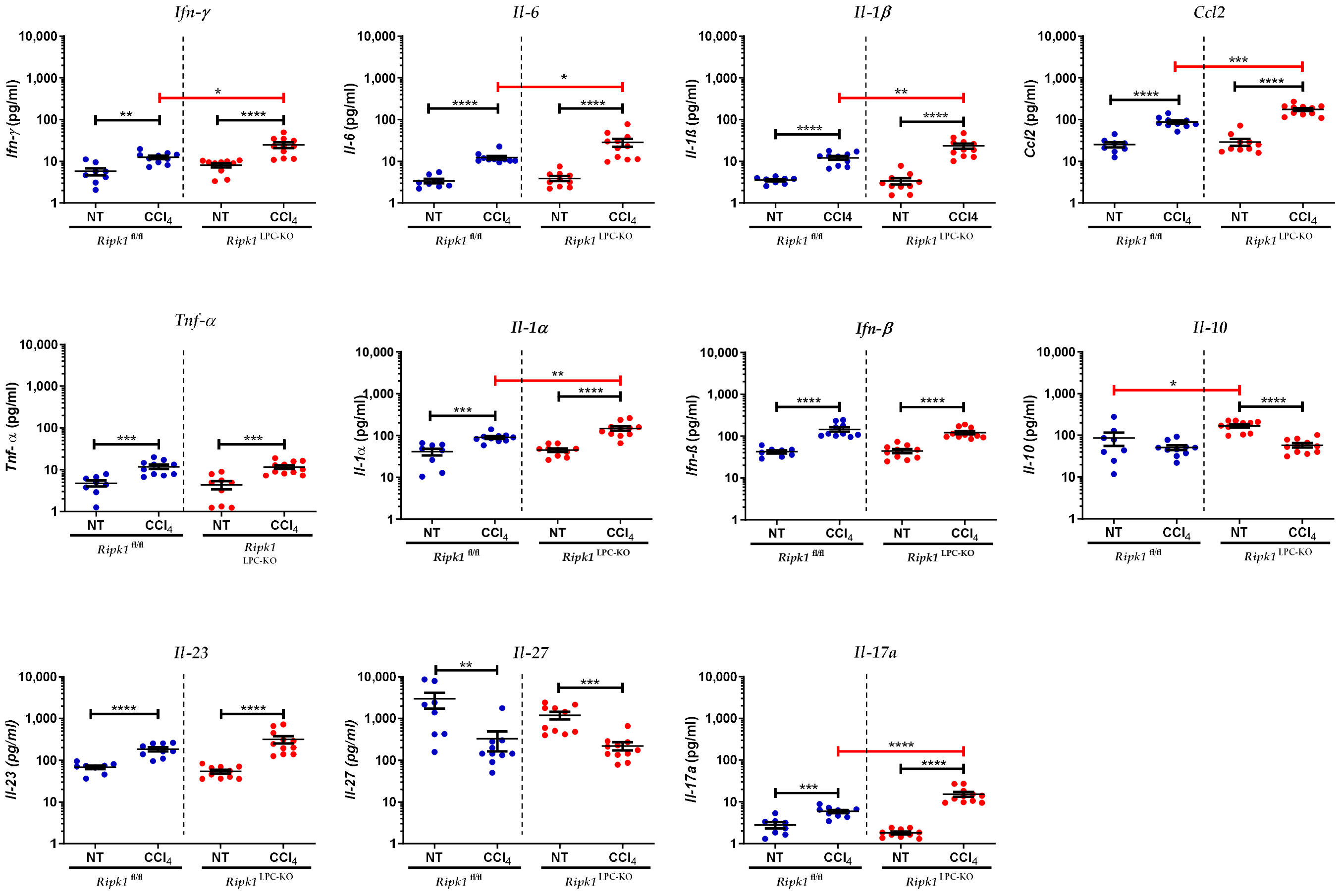

Figure S2: Serum levels of cytokines in non-treated and CCl_4-treated *Ripk1*$^{fl/fl}$ and *Ripk1*$^{LPC-KO}$ mice. *Ripk1*$^{fl/fl}$ and *Ripk1*$^{LPC-KO}$ mice were force-fed either with olive oil alone (control; NT for non-treated) or containing CCl_4 (dose of 2.4 g/kg body weight), 24 h before serum collection. Murine cytokines were captured by a bead-based immunoassay (BioLegend's LEGENDplex™ Mouse Inflammation Panel, multi-analyte flow assay kit) and quantified on a LSR X-20 Fortessa™ flow cytometer (BD Biosciences). For all graphs, each dot represents an individual and errors bars are expressed as means ±SEM (* $p < 0.05$; ** $p < 0.01$; *** $p < 0.001$ and **** $p < 0.0001$).

Figure S3

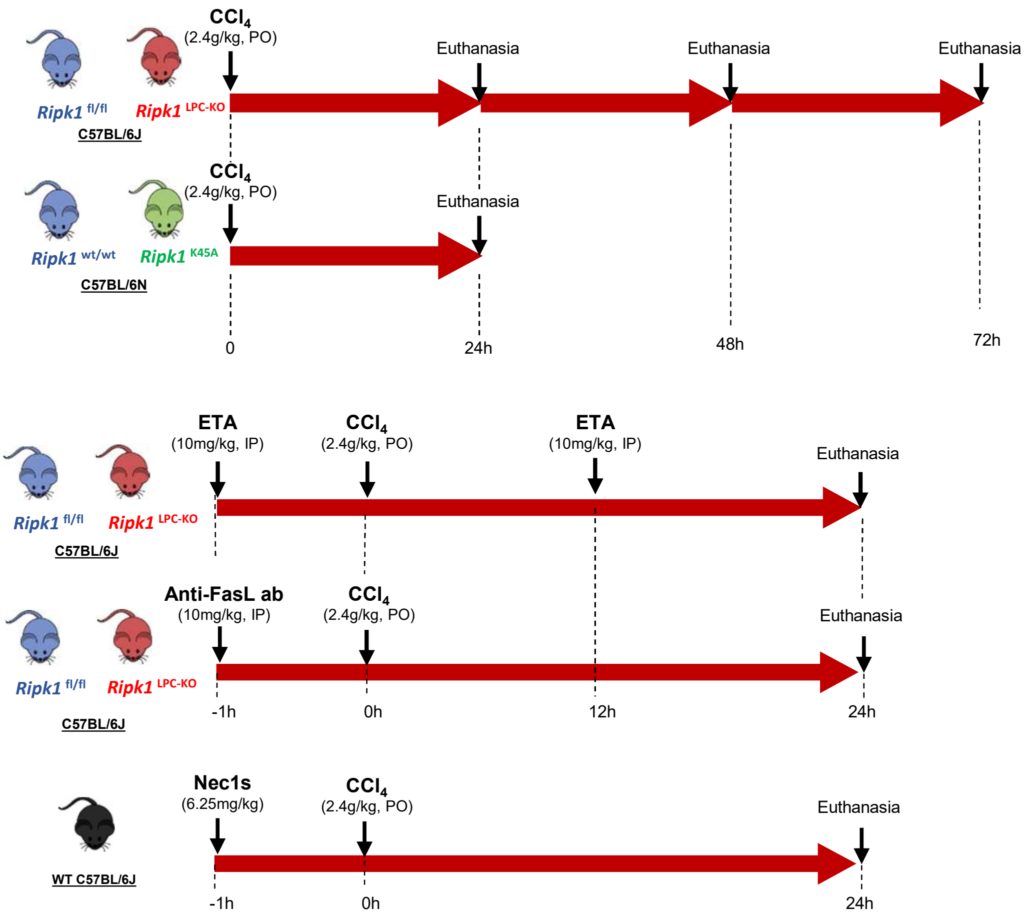

LPC : Liver parenchymal cells
K45A : Kinase Dead (KD)

Figure S3: Experimental design. *Ripk1*$^{LPC\text{-}KO}$ (red; LPC-KO, Liver Parenchymal Cell – Knock Out) and *Ripk1*^{K45A} (green; Kinase Dead, KD) C57BL/6 mice along with their respective WT littermates (blue; *Ripk1*$^{fl/fl}$ [fl, floxed] and *Ripk1*$^{wt/wt}$ [wt, wild-type]) were force-fed with olive oil alone (non-treated, NT) or containing CCl_4 with a final administered dose of 2.4g/kg (PO, Per Os). When used, etanercept (ETA) or anti-FasL antibody (ab) or vehicle (PBS) were intraperitoneally (IP) injected at 10 mg/kg, 1 hour before CCl_4 treatment, and 12 hour later for a second dose of ETA. In another experiment, Nec-1s or vehicle (PBS-DMSO 6%) was intravenously injected at 6.25 mg/kg 1 hour before CCl_4 treatment. Analysis were conducted 12, 24, 48 or 72 hours post CCl_4 administration as shown in the diagram.

Figure S4

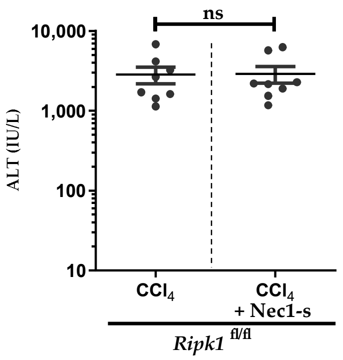

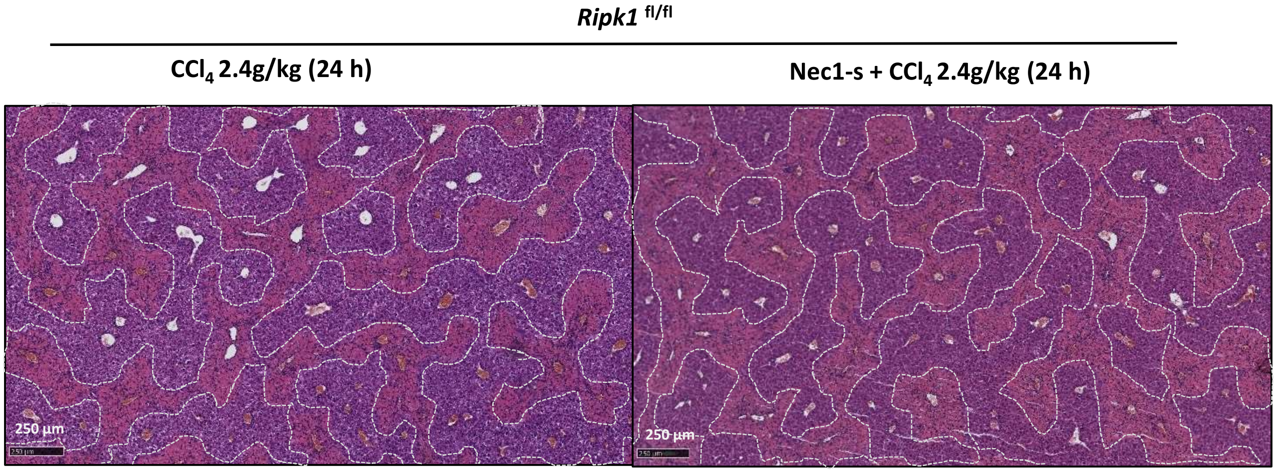

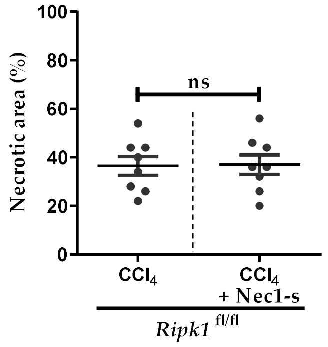

Figure S4: No contribution of RIPK1 kinase activity during CCl_4-induced hepatotoxicity. C57BL/6J *Ripk1*$^{fl/fl}$ mice were force-fed with olive oil containing CCl_4 (dose of 2.4 g/kg body weight), 24 hours before sample collection. Levels of serum alanine aminotransferase (ALT). Representative pictures of liver tissue sections stained by H&E with necrotic area quantifications. For all graphs, each dot represents an individual and errors bars are expressed as means ±SEM (ns, non-significant).

Table S1

Mouse Gene	Forward	Reverse
18S	5′-CGCCGCTAGAGGTGAAATTC-3′	5′-TTGGCAAATGCTTTCGCTC-3′
Hmox-1	5′-AGGTACACATCCAAGCCGAGA-3′	5′-CATCACCAGCTTAAAGCCTTCT-3′
Nfe2l2 (Nrf-2)	5′-TAGATGACCATGAGTCGCTTGC-3′	5′-GCCAAACTTGCTCCATGTCC-3′
Cybb (Nox-2)	5′-AGTGCGTGTTGCTCGACAA-3′	5′-GCGGTGTGCAGTGCTATCAT-3′
Nqo1	5′-AGGATGGGAGGTACTCGAATC-3′	5′-AGGCGTCCTTCCTTATATGCTA-3′
Il-6	5′-CGATGATGCACTTGCAGA-3′	5′-CTCTGAAGGACTCTGGCTTTG-3′
Ifn-γ	5′-AAGACAATCAGGCCATCAGC-3′	5′-CAGCAGCGACTCCTTTTCC-3′
Il-1b	5′-GCCACCTTTTGACAGTGATGAG-3′	5′-GACAGCCCAGGTCAAAGGTT-3′
Ccl2	5′-TCCCAATGAGTAGGCTGGAG-3′	5′-TCTGGACCCATTCCTTCTTG-3′
Rela (NFkBp65)	5′-GACCCCTGTCCTCTCACATCCG-3′	5′-CAGCTCCCAGAGTTCCGGTT-3′
Nfkbia (IkBα)	5′-TGAAGGACGAGGAGTACGAGC-3′	5′-TTCGTGGATGATTGCCAAGTG-3′
Tnfaip3 (A20)	5′-TGCCCAGTCTGTAGTCTTCG-3′	5′-AGTTGTTCAGCCATGGTCCT-3′
Cflar (cFLIP)	5′-GAGGCCAGCTCTCTTTTGCT-3′	5′-TCCACGGTTGCTTTGTCTGT-3′
Ikbk-γ (Nemo)	5-′GGTGGAGAGACTGAGCTTGG-3′	5′-CCTCTAAAGCTTGCCGATCC-3′
Saa1	5′-TGTTCACGAGGCTTTCCAAG-3′	5′-GTCCTCTGCCGAAGAATTCC-3′
Cxcl1	5′-CGCCTATCGCCAATGAGC-3′	5′-GAACCAAGGGAGCTTCAGG-3′
Ccl20	5′-TCTGCTCTTCCTTGCTTTGG-3′	5′-TCACCCAGTTCTGCTTTG-3′
Tnf-α	5′-TAGCTCCCAGAAAAGCAAGC-3′	5′-TTTTCTGGAGGGAGATGTGG-3′
Tnfrsf1a (TNFR1)	5′-ATCGAGAGGCTGGAGATGC-3′	5′-GGCGGGATTTCTCAGAGC-3′
Tnfrsf1b (TNFR2)	5′-CGCTGGTCTTCGAACTGC-3′	5′-CAGGAGGACACTTAGCACAGC-3′
Fas	5′-CTCCGAGTTTAAAGCTGAGG-3′	5′-TGTACTCCTTCCCTTCTGTGC-3′
Fasl	5′-GCAGCAGCCCATGAATTACC-3′	5′-GGTAATTCATGGGCTGCTGC-3′
Tnfsf10 (TRAIL)	5′-TGAGAACCTTTCAGGACACC-3′	5′-GGCCTAAGGTCTTTCCATCC-3′
Tnfrsf10b (TRAIL-R2)	5′-CCGGGCAGATCACTACACC-3′	5′-AGACAGGGTCCTCTTGATGG-3′

Table S1: Primer sequences
